# Supplementary material for: Integration of exome-seq and mRNA-seq using DawnRank, identified genes involved in innate immunity as drivers of breast cancer in the Indian cohort
Source: PeerJ. 2023 Oct 2;11:e16033. doi: 10.7717/peerj.16033 (PMC10552747; doi:10.7717/peerj.16033)
Supplement: Supplemental Information 1 — Additional data showing interactors and the variants [file peerj-11-16033-s001.docx]

**Supplementary Figures and Tables**


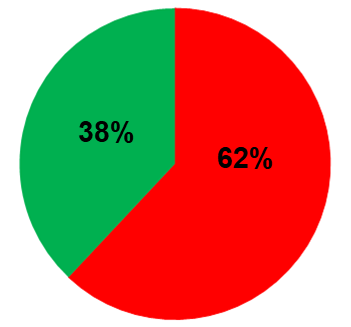

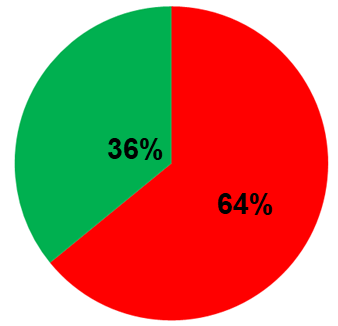

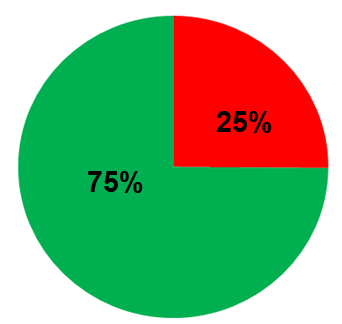

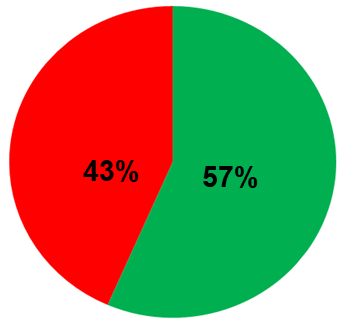


**d)**

**c)**

**b)**

**a)**

**Upregulation**

**Downregulation**

Supplementary Figure 1: Transcriptome Profile of each sample: Total % of DE genes upregulated and downreglated in each tumor sample (A) P1: EPH_1, (B)P2: EPH_2, (C) P3:TNBC, (D) P4: EP

**a)**


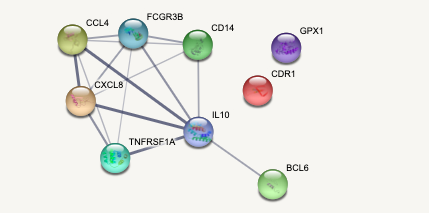

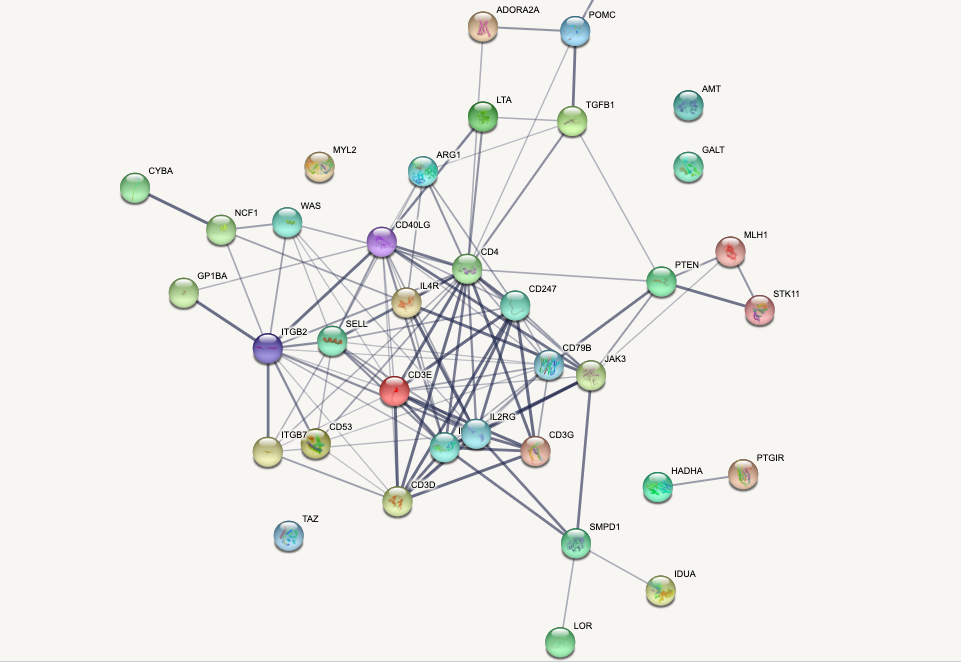


**b)**


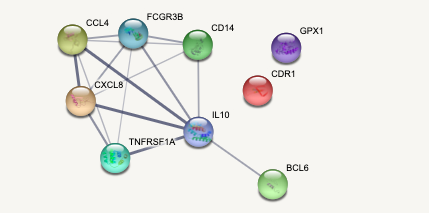

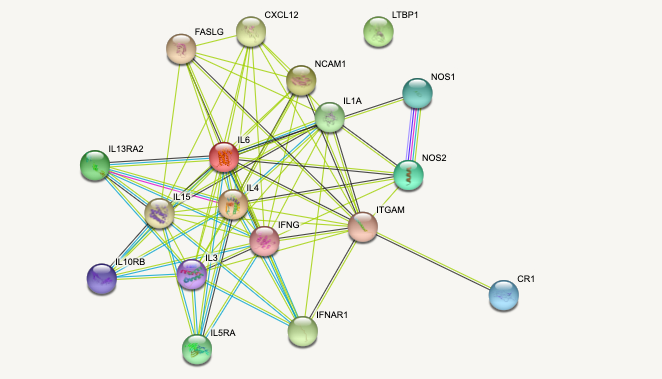


**d)**

**c)**

Supplementary Figure 2: STRING networks generated from gene lists from the WGCNA modules. a) EPH subtype, b) Estrogen positive subtype, c) Hmod subtype, d) TNBC subtype

| Gene | Mutation Type | Amino acid change | Number of Patients |
| --- | --- | --- | --- |
| TLR10 | missense | p.Asn241His | 2 |
| C7 | missense | p.Ser389Thr | 2 |
| BTK | missense | p.Glu24Gly | 1 |
| CARD8 | missense | p.Phe102Ile | 2 |

Supplementary Table 1a: List of genes and mutational burden in Immune system pathway and cytokine signaling pathway enriched in EPH subtype

| Gene | Mutation Type | Amino acid change | Number of Patients |
| --- | --- | --- | --- |
| IL4R | missense | p.Ile75Val | 2 |

Supplementary Table 1b: List of genes and mutational burden in T cell mediated immunity enriched in

Estrogen positive subtype

| Gene | Mutation Type | Amino acid change | Number of Patients |
| --- | --- | --- | --- |
| CCL4 | misense | p.Ser80Thr | 1 |
| FCGR3B | misense | p.Asn101Ser | 3 |
| GPX1 | misense | p.Pro200Leu | 1 |
| IFNAR1 | misense | p.Val168Leu | 2 |

Supplementary Table 1c: List of genes and mutational burden in Type II Immune response enriched in

Hmod subtype

| Gene | Mutation Type | Amino acid change | Number of Patients |
| --- | --- | --- | --- |
| C8A | misense | p.Ala36Glu | 1 |
| C8A | misense | p.Arg59Gln | 1 |
| C8A | misense | p.Gln93Lys | 2 |
| CR1 | misense | p.Asn1990Ser | 1 |
| CR1 | misense | p.His1658Arg | 1 |
| CR1 | misense | p.Ile1813Thr | 1 |
| CR1 | misense | p.Ile2065Val | 1 |
| CR1 | misense | p.Pro2277Arg | 1 |
| CR1 | misense | p.Thr1858Ala | 1 |
| CR1 | misense | p.Thr1858Met | 2 |
| CR1 | misense | p.Thr2060Ser | 3 |
| NCAM1 | misense | p.Phe7Leu | 3 |
| NOS1 | misense | p.Pro853Ser | 2 |
| NOS2 | misense | p.Arg1018Ser | 1 |
| NOS2 | misense | p.Ser608Leu | 1 |
| CR1 | misense | p.His1768Tyr | 1 |
| CR1 | misense | p.Thr2419Ala | 3 |
| IL5RA | misense | p.Ile129Val | 3 |
| ITGAM | misense | p.Ala859Val | 1 |
| ITGAM | misense | p.Pro1147Ser | 1 |
| LTBP1 | misense | p.Val1378Ala | 3 |

Supplementary Table 1d: List of genes and mutational burden in Interleukin and Interferon pathway

enriched in TNBC subtype
